# Standard versus accelerated riboflavin–ultraviolet corneal collagen crosslinking: Resistance against enzymatic digestion

**DOI:** 10.1016/j.jcrs.2015.10.004

**Published:** 2015-09

**Authors:** Nada H. Aldahlawi, Sally Hayes, David P.S. O'Brart, Keith M. Meek

**Affiliations:** From the Structural Biophysics Research Group (Aldahlawi, Hayes, Meek), School of Optometry and Vision Sciences, Cardiff University, Cardiff, and the Keratoconus Research Institute (O'Brart), Department of Ophthalmology, St Thomas Hospital, London, United Kingdom

## Abstract

**Purpose:**

To examine the effect of standard and accelerated corneal collagen crosslinking (CXL) on corneal enzymatic resistance.

**Setting:**

School of Optometry and Vision Sciences, Cardiff University, Cardiff, United Kingdom.

**Design:**

Experimental study.

**Methods:**

Sixty-six enucleated porcine eyes (with corneal epithelium removed) were assigned to 6 groups. Group 1 remained untreated, group 2 received dextran eyedrops, and groups 3 to 6 received riboflavin/dextran eyedrops. Group 4 had standard CXL (3 mW/cm^2^ ultraviolet-A for 30 minutes), whereas groups 5 and 6 received accelerated CXL (9 mW/cm^2^ for 10 minutes and 18 mW/cm^2^ for 5 minutes, respectively). Trephined central 8.0 mm buttons from each cornea underwent pepsin digestion. Corneal diameter was measured daily, and the dry weight of 5 samples from each group was recorded after 12 days of digestion.

**Results:**

All CXL groups (4 to 6) took longer to digest and had a greater dry weight at 12 days (*P* < .0001) than the nonirradiated groups (1 to 3) (*P* < .0001). The time taken for complete digestion to occur did not differ between the standard and accelerated CXL groups, but the dry weights at 12 days showed significant differences between treatments: standard CXL 3 mW > accelerated CXL 9 mW > accelerated CXL 18 mW (*P* < .0001).

**Conclusions:**

Standard and accelerated CXL both increased corneal enzymatic resistance; however, the amount of CXL might be less when accelerated CXL is used. The precise amount of CXL needed to prevent disease progression is not yet known.

**Financial Disclosure:**

No author has a financial or proprietary interest in any material or method mentioned.

Riboflavin–ultraviolet-A (UVA) corneal collagen crosslinking (CXL) is the first treatment modality shown to halt the progression of keratoconus^1^, ^2^, ^3^, ^4^ and other corneal ectatic disorders.^4^, ^5^, ^6^ The standard treatment protocol, which was first tested clinically by Wollensak et al.,^1^ involves the debridement of the central 7.0 mm of the cornea, followed by the application of riboflavin and a 30-minute exposure to 370 nm UVA at an energy of 3 mW/cm^2^. At this fluence, the procedure appears to be safe in terms of endothelial toxicity, provided the stromal corneal thickness is greater than 400 μm.^7^ In addition, beneficial clinical outcomes in terms of cessation of disease progression and improvements in visual and topographic parameters are consistently achieved,^1^, ^2^, ^3^, ^4^, ^5^, ^6^, ^8^ with reported follow-up of 4 to 6 years.^9^ However, the UVA exposure time required to achieve the wanted clinical effect,^10^ coupled with the need to instill riboflavin eyedrops for at least 20 to 30 minutes before irradiation (to achieve a homogeneous stromal uptake of riboflavin^11^), results in operative times in excess of 1 hour.

Recently, in an attempt to reduce patient treatment time, accelerated CXL protocols using higher fluences and shorter exposure times have been postulated. The envisaged safe and effective use of accelerated CXL is based on the Bunsen-Roscoe law of reciprocity,^12^ which predicts that the same subthreshold total cytotoxic corneal endothelial UVA dosage can be administered by increasing UVA fluence while simultaneously reducing exposure time. At present, published clinical studies of patients treated with accelerated CXL protocols are few; however, they report no adverse effects associated with accelerated treatment and a significant reduction in both topographic keratometry and corrected distance visual acuity at up to 46 months of follow-up.^13^, ^14^

Spoerl et al.^15^ demonstrated an increased resistance of the corneal stroma to enzymatic digestion after standard CXL, and this has since been replicated by others.^16^, ^17^ Because increased activity of proteinase enzymes and reduced activity of proteinase inhibitors have been identified in keratoconic corneas,^18^, ^19^ this increased resistance to proteinase digestion is liable to be an important factor in the protection against ectatic progression.^15^, ^17^ Therefore, to complement previously published studies focusing solely on the biomechanical changes in the cornea after CXL,^20^, ^21^ this study investigated the efficacy of standard and accelerated CXL protocols in terms of their ability to increase the resistance of the cornea against enzymatic digestion. Our previous studies have indicated that riboflavin–UVA causes the formation of crosslinks not only at the collagen fibril surface but also in the protein network surrounding the collagen.^16^ For this reason, pepsin was selected for this particular study because it is a nonspecific endopeptidase that can break down collagens and proteoglycan core proteins.

## Materials and methods

Sixty-six porcine eyes with clear intact corneas were obtained from a local European Community–licensed abattoir within 6 to 8 hours postmortem. By using a single-edged razor blade, the entire corneal epithelium was carefully removed from each eye. A detailed visual inspection was performed to confirm that the debridement technique had resulted in complete removal of the epithelium without damage to the underlying stroma. The central corneal thickness (CCT) of each eye was measured before and after epithelial debridement using a Pachette2 ultrasonic pachymeter (DGH Technology, Inc.). The 66 eyes were randomly and equally divided into the 6 treatment groups:

Group 1: Untreated controls receiving no eyedrops and no UVA exposure.
Group 2: Dextran-only controls receiving 20% dextran T500 eyedrops (Pharmacosmos A/S) every 5 minutes for 30 minutes and no UVA exposure.
Group 3: Riboflavin-only controls receiving riboflavin eyedrops (0.1% solution riboflavin-5-phosphate in 20% dextran T-500 solution, Mediocross D, Peschke Meditrade GmbH) every 5 minutes for 30 minutes and no UVA exposure.
Group 4: “Standard” 3 mW/cm^2^ CXL protocol (standard CXL 3 mW) receiving riboflavin 0.1% eyedrops in 20% dextran T-500 every 5 minutes for 30 minutes before exposure of the central 9.0 mm region of the cornea to UVA light with a fluence of 3 mW/cm^2^ for 30 minutes (CCL-365 Vario crosslinking system, Peschke Trade GmbH). Riboflavin eyedrops were reapplied at 5-minute intervals throughout the period of irradiation.
Group 5: Accelerated 9 mW/cm^2^ CXL protocol (accelerated CXL 9 mW) receiving riboflavin 0.1% eyedrops in 20% dextran T-500 every 5 minutes for 30 minutes, followed by a 10-minute exposure of the central 9.0 mm region to UVA light with a fluence of 9 mW/cm^2^ and reapplied riboflavin eyedrops at 5-minute intervals during the exposure.
Group 6: Accelerated 18 mW/cm^2^ CXL protocol (accelerated CXL 18 mW) receiving riboflavin 0.1% eyedrops in 20% dextran T-500 every 5 minutes for 30 minutes, followed by a 5-minute exposure of the central 9.0 mm region to UVA light with a fluence of 18 mW/cm^2^.

Immediately after treatment, the CCT was again measured. The cornea with a 4.0 to 5.0 mm scleral rim was then dissected from each globe, wrapped tightly in Clingfilm (to prevent moisture loss), and refrigerated until all treatments were complete. An 8.0 mm corneal button was trephined from the center of each cornea using a disposable skin biopsy punch (ref BP-80F, Kai Europe GmbH). The corneal buttons were weighed, then placed in individual plastic tubes, each containing 5 mL of pepsin solution, and incubated in a water bath (VwB6, VWR International bvba) at a temperature of 23°C. The pepsin solution was made of 1 g of 600 to 1200 U/mg pepsin from porcine gastric mucosa (Sigma-Aldrich Co. LLC) in 10 mL 0.1 M hydrochloric acid at pH 1.4. Previous studies^15^ have shown that changes in corneal disk thickness are not a reliable indicator of the rate of enzymatic digestion because of the considerable stromal swelling that occurs in the vertical direction within 24 hours of immersion in pepsin digest solution. Because the diameter of the anterior surface of each corneal button is unaffected by changes in stromal hydration,^15^ this parameter was used to monitor the rate of enzymatic digestion in 6 of the corneas from each treatment group. Measurements of anterior surface diameter were made using an electronic digital caliper (model CM145 4500360, Clarke International) at 24 hourly intervals until complete digestion had occurred. Because the diameter was found to vary slightly between different meridians of an individual specimen, the average of the major axis and minor axis diameter of each corneal button was recorded at each time point and statistically evaluated. The definition of complete digestion was the point at which the specimen could no longer be distinguished from the surrounding pepsin solution, even under microscopic examination.

To further assess the effect of each treatment on enzymatic resistance, 5 corneal disks from each group were removed from the pepsin digest solution after 12 days and placed in a 60°C oven until a constant dry weight was obtained. The average corneal dry weight (which represents the mass of undigested tissue) was calculated for each group.

### Statistical Analysis

Measurements of corneal thickness (before and after treatment), corneal disk diameter, dry weight, and complete digestion time were statistically analyzed using a 1-way analysis of variance test. Post hoc Bonferroni comparisons were used to isolate significant interactions. All statistical analyses were performed with Statistical Package for the Social Sciences software (SPSS Statistics 20, International Business Machines Corp.). A *P* value less than 0.01 was considered to be significant. Data are presented in the results as the mean ± standard deviation. The observed power computed using α equal to 0.05 was 1, demonstrating that the sample size was sufficient.

## Results

Measurements of CCT before and after epithelial removal and after each stage of treatment are shown in Table 1. No statistically significant differences in corneal thickness were observed between the groups either before or after epithelial removal. However, there was a significant reduction in corneal thickness after administration of dextran-containing solutions in both group 2 (20% dextran) and group 3 (riboflavin 0.1%–dextran 20%) (*P* < .0001). Application of the riboflavin solution (containing dextran) to the deepithelialized cornea (group 3) resulted in a significantly greater reduction in corneal thickness than application of the dextran-only solution (group 2) (*P* < .001). A significant reduction in corneal thickness was observed after CXL in groups 4, 5, and 6 (*P* < .0001). Because the post-treatment thickness of the irradiated corneas (groups 4, 5, and 6) did not differ from that of the nonirradiated riboflavin–treated corneas (group 3), the corneal thinning in CXL may be attributed to the application of riboflavin rather than to UVA exposure.

**Figure 1:**
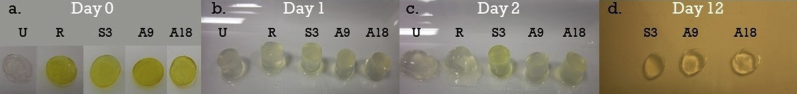
Corneal disks from left to right: untreated (*U*), riboflavin–dextran only (R), standard CXL 3 mW (S3), accelerated CXL 9 mW (A9), and accelerated CXL 18 mW (A18) before (*a*) and after 1 day (*b*), 2 days (*c*), and 12 days (*d*) of immersion in pepsin digest solution. All corneal buttons are swollen after 1 day in pepsin digest solution (*b*). After 2 days of digestion, the anterior curvature has been lost in the untreated corneas but remains intact in the crosslinked corneas (*c*). After 12 days, all nonirradiated buttons have been completely digested and only the anterior portion of the crosslinked corneas remain.

An approximately 10-fold increase in the thickness of the corneal disk, as a result of stromal swelling in the posterior–anterior direction, was observed in all corneal buttons within 24 hours of submersion in pepsin digest solution (Figure 1). After 1 week of digestion, the anterior portion of each treated and untreated corneal button had separated from the posterior portion. Once detached, the posterior stroma was rapidly digested (within 10 days); however, the anterior stromal button persisted considerably longer and maintained its form sufficiently to obtain reliable measurements of its changing diameter during the digestion process.

**Figure 2:**
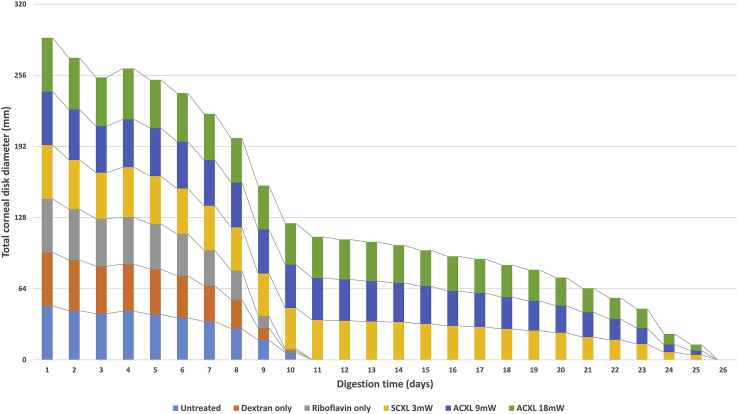
The summed diameter of all corneal disks (n = 8) in each treatment group shown as a function of time in pepsin digest solution (ACXL = accelerated crosslinking; SCXL = standard crosslinking).

Figure 2 shows the summed diameters of 6 corneal disks within each treatment group as a function of incubation time in pepsin solution. Statistical analysis revealed no significant difference in either the mean corneal button diameter of nonirradiated specimens (groups 1, 2, and 3) at any timepoint during digestion or in the time taken for complete digestion to occur (Table 2). Similarly, in the irradiated specimens, no significant difference in these parameters was detected between specimens treated with standard CXL (group 4) or accelerated CXL (groups 5 and 6). The diameter of the corneal disks in the CXL-treated groups (4, 5, and 6) was, however, significantly higher than that in the nonirradiated specimens (groups 1, 2, and 3) at all daily timepoints after 8 days (*P* < .0001) (Figure 2), and the time required for complete digestion to occur was significantly longer (*P* < .0001) (Table 2). By 12 days all nonirradiated corneas had been completely digested, but the mean diameter of the CXL-treated eyes (groups 4, 5, and 6) had decreased by only 27.2%, 27.0%, and 26.6% in the standard CXL 3mW, accelerated CXL 9mW, and accelerated CXL 18 mW groups, respectively.

At day 0, there was no significant difference between the mean wet weight of corneal disks in groups 1, 3, 4, 5, and 6 (*P* > .14). However, the mean wet weight of the dextran-only treated corneas (group 2) was significantly higher than that of the 3 mW standard CXL (group 4) (*P* < .03) and 9 mW accelerated CXL treated corneas (group 5) (*P* < .03) (Table 3).

Measurements of corneal disk dry weight after 12 days of digestion showed a statically significant difference between irradiated and nonirradiated corneas (*P* < .0001) and between irradiated corneas treated with 3 mW, 9 mW, or 18 mW accelerated CXL (*P* < .0001) (Table 3). The standard CXL 3 mW-treated corneas had a statistically higher mean dry weight than the 9 mW and 18 mW accelerated CXL-treated corneas (*P* < .0001) and the 9 mW accelerated CXL group had a higher dry weight than the 18 mW accelerated CXL group (*P* < .003) (Table 3).

## Discussion

Although the precise etiology of keratoconus is unknown,^22^ an increased activity of proteinase enzymes and a reduced activity of protease inhibitors have been identified in keratoconic corneas.^18^ This increased stromal protein digestion is thought to be an important factor in the resultant corneal thinning and biomechanical instability seen in keratoconic eyes.^19^ Spoerl et al.^15^ demonstrated an increased resistance of corneal stromal tissue to enzymatic digestion after CXL, with irradiances of 2 mW/cm^2^ and 3 mW/cm^2^ UVA. This increased resistance to proteinase digestion after CXL has been replicated by others^16^, ^17^ and is likely to be an important factor in preventing disease progression.^15^, ^19^

In this study, the enzymatic resistance of nonirradiated-treated porcine corneas was compared with that of standard CXL- and accelerated CXL-treated corneas. Similar to other clinical- and laboratory-based studies,^16^, ^23^, ^24^ a significant reduction in corneal thickness was observed after CXL using an isotonic riboflavin solution. Because the posttreatment thickness of the irradiated corneas did not differ from that of the nonirradiated riboflavin-treated corneas, the corneal thinning observed during CXL and accelerated CXL may be attributed predominantly to the application of riboflavin–dextran solution rather than to the effect of CXL after UVA exposure. The application of riboflavin solution (containing 20% dextran) resulted in a significantly greater reduction in corneal thickness than the application of the same concentration of dextran in the absence of any riboflavin. This finding may be the result of riboflavin increasing the ionic strength of the applied solution, because higher ionic strengths are known to be associated with lower corneal hydrations^25^ and reduced corneal thickness.

As described previously,^15^, ^16^ significant stromal swelling occurred (predominantly in the posterior stroma) in all corneal buttons during the first 24 hours in pepsin solution. This observation can be attributed to the negatively charged glycosaminoglycan components of the proteoglycans within the extracellular matrix, which result in the pepsin digest solution being drawn into the tissue.^26^ The higher ratio of keratan sulfate to chondroitin sulfate in the posterior stroma compared with the anterior^27^ may explain why most of the swelling occurred in this region because keratan sulfate has a higher water affinity than chondroitin sulfate.^28^ Interestingly, the separation of the cornea into anterior and posterior stromal regions during the first week of digestion was observed in all treated and untreated corneas. The separation of the corneal buttons cannot, therefore, be attributed to CXL-induced changes within the anterior stroma but must instead be the result of naturally occurring structural differences that exist between the anterior and posterior regions. The diameter of the anterior portion of the corneal button was unaffected by the changes in corneal hydration and, therefore, formed a much more reliable measure of the rate of enzymatic digestion than measurements of corneal thickness. However, calculations based on the sample size used and the standard deviation of diameter measurements confirmed that the sensitivity of the technique was such that differences between groups (in terms of the time taken for complete digestion) of less than 1 day could not be detected by this method. For this reason, measurements of corneal disk dry weight (which reflect the mass of undigested corneal tissue) were recorded at day 12 of the digestion process to allow more subtle differences in enzymatic resistance between treatment groups to be identified.

Our results showed an increased resistance to proteinase digestion after standard CXL that is in agreement with the findings of other investigators.^15^, ^16^, ^17^ However, for the first time, we have shown that a similar increase in enzymatic resistance can also be achieved using higher fluences (up to 18 mW/cm^2^) and shorter exposure times. Although the diameter measurements detected no difference in enzymatic resistance between the CXL protocols used in this study, the mean dry weight of corneal tissue after 12 days of protein digestion was found to differ significantly between groups (3 mW accelerated CXL > 9 mW accelerated CXL > 18 mW accelerated CXL). Measurements of corneal dry weight, which represent the total mass of undigested tissue and negate the complications associated with within-sample variations in corneal thickness and between-sample differences in hydration, provide a more accurate means of assessing the relative efficacy of CXL procedures at increasing corneal enzymatic resistance. Our findings suggest that in protocols that use a higher fluence and shorter exposure time, either the most anterior layers of the corneal stroma may be crosslinked equally and the effective depth of CXL is reduced or the intensity of CXL (which is known to be depth dependent^29^) decreases more rapidly as a function of depth. One interesting observation in previous studies is the presence of a shallower demarcation line in accelerated CXL compared with standard CXL,^30^, ^31^ suggesting that this may represent a reduced or shallower CXL effect. However, this assumes that the depth of the demarcation line correlates directly with the degree and depth of CXL, and currently there is no direct evidence to support this. The so-called stromal demarcation line, first described by Seiler and Hafezi,^32^ has been shown to possibly be shallower in older patients and those with more severe ectatic disease.^33^ It has been found to be thicker centrally and thinner peripherally^34^ and possibly related to an increased density of the extracellular matrix.^35^ Although a deeper demarcation line has been associated with a larger decrease in corneal thickness,^36^ its depth has not been shown to be correlated to either visual or keratometric changes 6 months postoperatively.^33^ It may simply represent natural wound-healing responses rather than delineate the true area between crosslinked and uncrosslinked tissue, and more research is required to ascertain the true nature of this demarcation line and its relationship to the actual CXL process.

Other studies have concentrated on comparing the biomechanical changes after CXL and accelerated CXL using methods such as scanning acoustic microscopy and extensiometry. Hammer et al.^37^ reported a reduced corneal stiffening effect with increasing UVA intensity (up to 18 mW); however, others have shown similar biomechanical changes after both standard 3 mW/cm^2^ CXL and 9 to 10 mW/cm^2^ accelerated CXL,^20^, ^21^ but a sudden decrease in efficacy with high intensities (greater than 45 mW/cm^2^).^38^ The failure of the Bunsen-Roscoe law^12^ of reciprocity in cases of high intensity and short illumination time is not yet understood but may be related to rapid oxygen consumption and subsequent reduced oxygen availability, which has been shown to limit the photochemical CXL process.^39^ Oxygen and its vital role in free radical production has been shown to be central in driving the CXL process.^39^ Therefore, limitations in availability because of reduced time to replenish suitable oxygen levels can theoretically inhibit the photochemical CXL process.^39^ In our study, we found only subtle differences in enzymatic resistance, with increasing UVA intensity up to 18 mW/cm^2^. Further studies with energies of 30 mW/cm^2^ and above are indicated to see whether the results of pepsin digestion studies replicate those of extensometry and other mechanical methods.

Even though most laboratory results are supportive of accelerated CXL, published clinical studies of the technique are limited. A significant reduction in topographic keratometry and improvement in corrected distance acuity, comparable to standard CXL, have been reported at the 6-month follow-up.^13^ In a randomized prospective study comparing a fluence of 7 mW/cm^2^ for 15 minutes with 3 mW/cm^2^ for 30 minutes,^14^ similar clinical results for ectasia stabilization were reported after each treatment protocol, and neither treatment resulted in any adverse effects. Similarly, clinical studies using 9 mW/cm^2^ for 10 minutes have shown a significant reduction in keratometry after CXL, with no adverse effects in terms of endothelial cell counts at 3 months.^40^ More recently, it was shown that accelerated CXL with an irradiance of 30 mW/cm^2^ for 3 minutes resulted in a significant improvement in uncorrected distance visual acuity and a reduction in keratometry at 6 months.^41^ This finding was supported by a nonrandomized study comparing standard CXL with 30 mW/cm^2^ accelerated CXL, which found no difference in visual, refractive, keratometric, or biomechanical parameters between the 2 treatments at the 12-month follow-up.^42^

Although our studies have indicated that the amount of CXL may be less with accelerated CXL, the minimum effective amount of CXL needed for ectasia stabilization has not yet been established. The success of the clinical studies described previously indicates that the amount of CXL produced by accelerated CXL may be sufficient to prevent keratoconus progression. Clearly, further clinical studies, especially randomized prospective trials, will be necessary to ascertain the clinical safety and efficacy of accelerated CXL. But thus far, the accumulating clinical and laboratory evidence demonstrates its similar efficacy to standard CXL, and its clear benefits in terms of patient and surgeon convenience support its use.

**What Was Known**
• Standard riboflavin–UVA CXL increases both the strength of the cornea and its resistance to enzymatic digestion and has proved to be successful in halting keratoconus progression. The effect of accelerated CXL protocols on corneal enzymatic resistance is currently unknown.

**What This Paper Adds**
• Both standard and accelerated riboflavin–UVA corneal CXL protocols (up to 18 mW) resulted in an increase in corneal enzymatic resistance.
• Differences in enzymatic resistance suggest that the accelerated protocols result in less CXL than the standard treatment.

## Figures and Tables

**Table 1:** Central corneal thickness before and after epithelial removal and after treatment.∗

Groups	Central Corneal Thickness			
Before Epithelial Removal (μm)∗	After Epithelial Removal (μm)∗	Posttreatment (μm)∗	Change (%)†
Untreated	830 ± 46	739 ± 45	NA	NA
Dextran only	821 ± 41	721 ± 39	602 ± 41	−17
Riboflavin–dextran only	775 ± 45	676 ± 45	508 ± 39	−24.9
SCXL 3 mW	792 ± 48	694 ± 50	494 ± 28	−28.8
ACXL 9 mW	820 ± 38	716 ± 40	514 ± 31	−28.1
ACXL 18 mW	807 ± 32	706 ± 34	495 ± 34	−29.9

ACXL = accelerated crosslinking; NA = not applicable; SCXL = standard crosslinking

∗ Data are given as mean ± standard deviation.

† Percentage change from corneal thickness postepithelial removal to posttreatment

**Table 2:** Time taken for the complete digestion of treated and untreated corneal buttons.

Group	Time Taken for Complete Digestion (Days)		
Minimum	Maximum	Mean ± SD
Untreated	10	11	10.5 ± 0.55
Dextran only	10	11	10.5 ± 0.55
Riboflavin–dextran only	9	11	9.83 ± 0.75
SCXL 3 mW	24	26	24.7 ± 1.03
ACXL 9 mW	23	26	24.7 ± 1.03
ACXL 18 mW	24	27	24.8 ± 0.98

ACXL = accelerated crosslinking; SCXL = standard crosslinking

**Table 3:** Corneal disk weight before and after 12 days of pepsin digestion.

Group	Day 0 (Wet Weight) (g)∗	Day 12 (Dry Weight) (g)∗
Untreated	0.0540 ± 0.0050	0 ± 0
Dextran only	0.0563 ± 0.0055	0 ± 0
Riboflavin–dextran only	0.0477 ± 0.0047	0 ± 0
SCXL 3 mW	0.0438 ± 0.0032	0.0041 ± 0.0013
ACXL 9 mW	0.0440 ± 0.0038	0.0020 ± 0.0005
ACXL 18 mW	0.0445 ± 0.0018	0.0008 ± 0.0003

ACXL = accelerated crosslinking; SCXL = standard crosslinking

∗ Data are given as mean ± standard deviation.
